# Advantages and Limitations of Current Imaging Techniques for Characterizing Liposome Morphology

**DOI:** 10.3389/fphar.2018.00080

**Published:** 2018-02-06

**Authors:** Annie-Louise Robson, Paul C. Dastoor, Jamie Flynn, William Palmer, Antony Martin, Doug W. Smith, Ameha Woldu, Susan Hua

**Affiliations:** ^1^School of Biomedical Sciences and Pharmacy, University of Newcastle, Callaghan, NSW, Australia; ^2^Centre for Organic Electronics, University of Newcastle, Callaghan, NSW, Australia; ^3^Hunter Medical Research Institute, New Lambton Heights, NSW, Australia; ^4^School of Environmental and Life Sciences, University of Newcastle, Callaghan, NSW, Australia

**Keywords:** liposomes, microscopy, imaging, nanoparticles, light microscopy, electron microscopy, atomic-force microscopy

## Abstract

There are currently a number of imaging techniques available for evaluating the morphology of liposomes and other nanoparticles, with each having its own advantages and disadvantages that should be considered when interpreting data. Controlling and validating the morphology of nanoparticles is of key importance for the effective clinical translation of liposomal formulations. There are a number of physical characteristics of liposomes that determine their *in vivo* behavior, including size, surface characteristics, lamellarity, and homogeneity. Despite the great importance of the morphology of nanoparticles, it is generally not well-characterized and is difficult to control. Appropriate imaging techniques provide important details regarding the morphological characteristics of nanoparticles, and should be used in conjunction with other methods to assess physicochemical parameters. In this review, we will discuss the advantages and limitations of available imaging techniques used to evaluate liposomal formulations.

## Introduction

**FIGURE 1:**
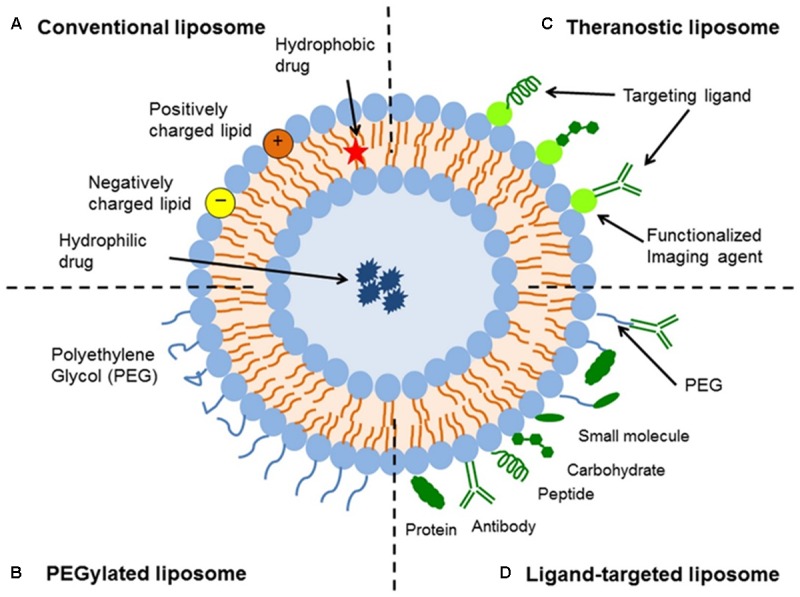
Schematic representation of the different types of liposomal drug delivery systems – **(A)** Conventional liposome, **(B)** PEGylated liposome, **(C)** Ligand-targeted liposome, and **(D)** Theranostic liposome (Sercombe et al., 2015).

Liposomes are a type of nanocarrier that have been widely investigated for drug-delivery purposes. They are composed of phospholipid bilayers which enclose a distinct aqueous space, thereby allowing encapsulation of both hydrophilic and hydrophobic compounds (Metselaar and Storm, 2005). Liposomes are able to stabilize therapeutic compounds and overcome barriers to cellular and tissue uptake (Ding et al., 2006; Hua and Wu, 2013). This allows them to improve targeting of compounds to sites of disease and consequently reduce accumulation in non-target organs (Bakker-Woudenberg et al., 1994; Mastrobattista et al., 1999; Hua, 2013; Hua et al., 2015; Sercombe et al., 2015; Zununi Vahed et al., 2017). There are four main types of liposomes based on their surface characteristics – conventional liposomes, PEGylated liposomes, ligand-targeted liposomes, and theranostic liposomes (**Figure 1**; Sercombe et al., 2015). Ligand-targeted liposomes provide the potential for site-specific delivery of drugs to certain tissues or organs that selectively express the targeted ligand (Willis and Forssen, 1998; Bendas, 2001; Sawant and Torchilin, 2012), whereas PEGylated liposomes confer steric hindrance to enhance the circulation half-life of the delivery system following systemic administration (Torchilin, 1994; Wang et al., 2015). Liposomes incorporating a combination of the various delivery platforms can further improve the delivery of encapsulated compounds, depending on the route of administration and site of disease.

Following the manufacturing process, liposomes are characterized to ensure homogeneity across a number of parameters, including drug encapsulation, ligand-conjugation, lipid composition, surface charge, and morphological properties (e.g., size, shape, and number of lamellae) (Kuntsche et al., 2011). These characteristics are important as they can have a major impact on the behavior of liposomes both *in vitro* and *in vivo* (Sawant and Torchilin, 2012; Sercombe et al., 2015). Recognition and clearance of liposomes by the body’s defenses, including the reticuloendothelial system (RES) and adsorption of opsonins with subsequent uptake by the mononuclear phagocytic system, are major contributors to the clearance and degradation of liposomes (Senior, 1987; Cullis et al., 1998; Ishida et al., 2001). Therefore, being able to determine the physicochemical properties of manufactured liposomes is important to optimize a formulation for further translational evaluation.

A major aspect in the physicochemical assessment of liposomes is visualizing the morphology of the nanoparticles using microscopy. There are a number of techniques available for imaging liposomes and other nanoparticles that can be broadly categorized into light, electron, or atomic-force microscopy (Bibi et al., 2011). Each technique has its own advantages and limitations, which should be considered when evaluating studies on nanoparticle-based drug delivery systems **(Table 1)**. This review will evaluate each imaging technique used to assess the morphological characteristics of liposomes.

**Table 1:** Summary of current imaging techniques for characterizing liposome morphology.

Technique	Advantages	Disadvantages
Basic light microscopy	• Rapid and simple	• Unable to provide comprehensive information about the lipid bilayer, especially for SUVs
	• Provides general information on the size, shape, homogeneity, and degree of aggregation, particularly for GUVs	
Polarization microscopy	• Provides an alternative method to confirm the formation of vesicles	• Unable to provide conclusive observations regarding the bilayer characteristics or lamellarity of the vesicles
	• Optimal clarity for large vesicles in the micrometer range	
Fluorescence microscopy	• Especially useful when viewing GUVs, where information can be obtained regarding the shape, size, and fluidity of the lipid vesicles	• Addition of probes and dyes can potentially interfere with the properties of the lipid vesicles and/or cause experimental artifacts, resulting in inaccurate data interpretation
	• Can apply multiple probes within a sample to provide information about the membrane structure itself	• Photo-induced lipid peroxidation can lead to domain formation
		• Prolonged exposure to fluorescent light can result in bleaching and loss of fluorescence intensity
Confocal microscopy	• Superior image clarity over fluorescence microscopy	• Unable to produce high definition images of SUVs or oligolamellar liposomes
	• Can provide a composite 3D image of the sample	
	• Capable of visualizing the internal structure of lipid vesicles, particularly for GUVs	
Scanning electron microscopy	• Allows visualization of small vesicles under very high magnification	• Unable to provide detailed information on the lamellarity and internal structure of lipid vesicles
	• Provides general detail on the size and spherical morphology of lipid vesicles	• Liposome structure may suffer perturbations due to the high-vacuum conditions and staining processes required prior to imaging
Transmission electron microscopy *Negative staining technique*	• Provides much higher magnification for imaging nanoparticles, including SUVs	• Vesicles are in direct contact with the grid, which may affect their orientation and morphology
	• Provides information on morphology, size distribution, homogeneity, and surface structure	• Placing the sample under vacuum can cause further dehydration of the sample
		• Sample preparation can cause changes to the original liposome structure and lead to the creation of light and dark fringes that may be mistaken for lamellar structures
Transmission electron microscopy *Freeze–fracture technique*	• Provides much higher magnification for imaging nanoparticles, including SUVs	• Artifacts may still occur in the sample during preparation due to insufficient freezing rate, re-deposition of solvent molecules and/or mechanical stress
	• Does not require any drying process	
	• Provides detailed information on the 3D structure of the vesicles and bilayer organization	
	• Replicas closely reflect the original native state of the sample	
Transmission electron microscopy *Cryogenic TEM*	• Most useful form of microscopy currently available to study liposome	• Utilizes lower doses of electrons, which often results in lower resolution compared to other TEM methods
	• Allows for the analysis of liposomes in their most native state	• Artifacts are still possible due to the formation of a thin film of amorphous ice and the use of blotting on the sample applying shear forces during the film formation
	• Avoids issues with chemical fixation, dehydration, cutting, and staining	
	• Provides detailed information on the size, shape, internal structure, and lamellarity of liposomes	
	• Sample preparation minimizes the formation of ice crystals and preserves proteins or other materials	
	• Resolution range is ∼5 to 500 nm, as defined by the thickness of the film	
Environmental scanning electron microscopy	• Allows visualization of small vesicles under very high magnification	• Unable to provide detailed information on the internal structure of lipid vesicles
	• Provides general information on the size and shape of lipid vesicles	
	• Allows imaging of dynamic changes of wet systems without previous sample preparation	
	• Does not require the use of fixing, staining or freezing of vesicles	
	• Able to modify sample environment, including pressure, temperature and gas compositions	
Atomic force microscopy	• Outstanding resolution in the order of fractions of a nanometer	• Requires nanoparticles to be adsorbed onto support surfaces, which can modify the size and shape of the vesicles
	• Provides 3D imaging of liposomes and details on morphology, size distribution, homogeneity, stability, and surface structure	• Periodic contact of the probing tip can drag the liposomes as it moves across the vesicles in a sample
	• Does not need to operate in a vacuum and can operate in ambient air or under liquid	
	• Can provide information about the mechanical and chemical properties of a sample surface through force measurements	

## Light Microscopy

Light or optical microscopy refers to microscopes that utilize visible light and an arrangement of lenses to magnify a field of view (Murphy and Davidson, 2012c). Basic light microscopy itself is unable to provide comprehensive information about the lipid bilayer compared to the detail offered by other microscopy techniques. However, it can be used to rapidly obtain an image of vesicles using basic laboratory equipment (Bibi et al., 2011). This technique can be particularly useful when gathering general information on the size, shape, homogeneity, and degree of aggregation of a liposome sample (Nallamothu et al., 2006). Light microscopes have an ultimate resolution of ∼250 nm (governed by the smallest diffraction-limited spot size that can be achieved by the instrument) and, as such, are typically incapable of providing detailed information regarding the structures of small unilamellar vesicles (SUVs) and the lamellarity of vesicles (Bibi et al., 2011). Generally, light microscopy can only provide significant information on giant unilamellar vesicles (GUVs), which can range from single to hundreds of micrometers in diameter (Bagatolli, 2009). Incorporation of fluorescent probes, polarization techniques, and application of high-resolution confocal microscopy can provide more information about the 3D structure and lamellarity of the vesicles (Bagatolli, 2009).

### Polarization Microscopy

This type of light microscopy utilizes the unique ability of polarized light to interact with polarizable bonds of ordered molecules (Murphy and Davidson, 2012d). Enhanced light absorption occurs when molecular dipoles in the sample are aligned with the polarization vector of the incident light wave; resulting in phase differences between sampling light rays, which in turn produce interference-dependent changes in amplitude in the image plane (Murphy and Davidson, 2012d). Image contrast then arises not only from the effects of interference and diffraction, but also due to the presence of ordered molecular arrangements (Murphy and Davidson, 2012d). It can be used to study the form and dynamics of many ordered cellular structures, including lipid bilayers of plasma membranes (Bibi et al., 2011; Murphy and Davidson, 2012d). Images can be obtained in either monochrome or color. This technique provides an alternative method to visualize liposomes, particularly to confirm the formation of vesicles. It does not provide conclusive observations regarding the bilayer characteristics or lamellarity of the vesicles (Bibi et al., 2011). In addition, polarization microscopy is also limited by the size of the vesicles that can be visualized, with large vesicles in the micrometer range having the optimal clarity. This technique does not provide clear images of particles in the nanometer range.

### Fluorescence Microscopy

Fluorescence or “epifluorescence” microscopy is a special form of light microscopy that exploits the ability of fluorochromes to emit light after being excited with light of a certain wavelength (Murphy and Davidson, 2012b). This technique is widely used in biophysics to provide essential information about the structure and dynamics of membrane components (Bouvrais et al., 2010). In these studies, fluorescent probes are specifically incorporated into the membrane under investigation, permitting visualization of the structure. A large range of fluorescent dyes with various chemical and photonic properties are commercially available, catering to a variety of research questions. For example, certain fluorescent dyes may prefer specific membrane environments exhibiting different arrangements and lateral packing (Bouvrais et al., 2010). As such, fluorescence microscopy allows for the potential to simultaneously apply multiple probes within a sample to provide information about the membrane structure itself. The fluorescent probes can be placed within the aqueous compartment as well as the lipid bilayer of liposomes (Bibi et al., 2011). This arrangement can be especially useful when viewing GUVs, where information can be obtained regarding the shape, size and fluidity of the lipid vesicles (Klymchenko et al., 2009; Bouvrais et al., 2010). Incorporation of probes, such as rhodamine-labeled lipids, directly into the lipid bilayer can also allow visualization of the lamellarity of liposomes (Bibi et al., 2011).

The disadvantage of fluorescent microscopy is that the addition of probes and dyes to a membrane system can potentially interfere with the properties of the liposomal delivery system (Bouvrais et al., 2010; Bibi et al., 2011; Murphy and Davidson, 2012b). However, the use of low dye concentrations (≤1 mol%) has been shown to have minimal impact on the physical properties of the membrane (Bouvrais et al., 2010). It is also important to note that the choice of fluorescent dye is a critical step, as some dyes can induce large changes in the host membrane and/or cause experimental artifacts, resulting in inaccurate data interpretation (Bouvrais et al., 2010). In addition, photo-induced lipid peroxidation can also lead to domain formation even in simple dye systems. This process can result in the formation of large rafts and spontaneous facet formation (Bouvrais et al., 2010). Prolonged exposure to fluorescent light can also result in bleaching and loss of fluorescence intensity. Therefore, the choice of fluorescent dye and the development of new membrane probes are important considerations when using fluorescence microscopy (Klymchenko et al., 2009).

### Confocal Microscopy

Confocal scanning microscopy has been an advancement in the area of fluorescence microscopy. Rather than illuminating the entire sample, an image is built by scanning one (or more) focused beams of light across the sample. Light returning from the illuminated sample passes through an aperture that rejects out-of-focus light from above and below the plane of interest; ensuring that only images from a small depth of field are obtained, greatly improving the out-of-plane resolution (Bibi et al., 2011; Murphy and Davidson, 2012a). Using this technique, a “z-stack” of images is collected, starting from the top of the vesicle followed by images taken in defined z-increments to the bottom of the sample, resulting in a composite 3D image of the sample (Bibi et al., 2011; Murphy and Davidson, 2012a). Confocal scanning microscopy has become a more attractive technique over epifluorescent light microscopes due to its superior image clarity. In the case of GUVs, this technique is capable of visualizing the internal structure of the lipid systems, which is often not possible with other microscopy methods (Ruozi et al., 2011; Mertins and Dimova, 2013). For example, separation of the aqueous and lipid bilayer phase can be clearly visualized in larger vesicles (Mertins and Dimova, 2013). However, confocal microscopy is still diffraction-limited and, therefore, unable to produce high-definition images of SUVs or oligolamellar liposomes (Ruozi et al., 2011).

## Electron Microscopy Techniques

Electron microscopy is a method for the visualization of vesicles under very high magnification (Henry, 2005). It is widely used in the characterization of lipid vesicles as the electron wavelength (and hence diffraction-limited resolution), is many orders of magnitude lower than that of optical microscopy, and therefore provides super-resolution for clear visualization of small liposomes (Bibi et al., 2011; Ruozi et al., 2011). This technique uses a beam of electrons focused onto the surface of the sample by various electromagnetic lenses. The electrons are then scattered by the sample, and are then refocused and magnified by a further series of electromagnetic lenses in the imaging column to produce a projected image (Henry, 2005). There are a number of different types of electron microscopes, each requiring a different sample preparation method.

### Scanning Electron Microscopy (SEM)

Scanning electron microscopes (SEMs) utilize an electron beam that is scanned across or over a sample (rather than through a sample) to produce a magnified image of an object (Adler and Schiemann, 1985). Alder et al. first attempted to use SEM to characterize liposomes in 1984 (Adler and Schiemann, 1985). They showed that using the freeze-drying method to prepare the liposome samples for SEM resulted in a large proportion of visible lumps and crusted material (Adler and Schiemann, 1985). More recent studies have shown that the liposome structure itself may suffer perturbations due to the high-vacuum conditions and staining processes required for this preparation technique (Ruozi et al., 2011). SEM is now not commonly used for analyzing liposomes because it requires the sample to be dried or fixed prior to imaging (Ruozi et al., 2011). However, SEM can provide general information on the concentric structure of the different lipid layers, as well as give detail on the size and spherical morphology of a preparation (Nirale et al., 2009).

### Transmission Electron Microscopy (TEM)

Transmission electron microscopy (TEM) is the most frequently used imaging method for the evaluation of the structure of nanoparticles (Henry, 2005; Kuntsche et al., 2011). It involves the preparation of a thin sample (<100 nm thick) that is placed in a vacuum chamber. The electron beam crosses the sample, where it is then focused by the objective lens to form an image. TEM can readily image soft-matter samples with a spatial resolution down to well below 1 nm in size (Henry, 2005; Kuntsche et al., 2011; Ruozi et al., 2011). This technique can be further categorized based on the sample-preparation method utilized – in particular, negative staining, freeze–fracture and cryogenic TEM. It can provide information on surface modifications of nanoparticles as it provides better contrast and contour of images than other microscopy techniques. For example, conjugation of transferrin to the surface of DSPC/cholesterol liposomes showed a particulate surface coating with negative-stain TEM, which was absent in the unconjugated liposome preparations (Anabousi et al., 2005). In a separate experiment, specific antibodies raised against human transferrin were added before the negative stain procedure to confirm the identity of the particulate surface coating (Anabousi et al., 2005). Therefore, TEM provides the possibility to achieve much-improved resolution when assessing the conjugation of ligands to the surface of liposomes.

#### Negative Staining Technique

In negative-stain TEM, sample preparation involves a small amount of hydrated sample being placed onto a grid. As the vesicles are in direct contact with the grid, their orientation and morphology may be affected, and hence this should be taken into account (Bibi et al., 2011). It is also necessary to embed the liposomes in a suitable electron-dense material (e.g., heavy metal salts like uranyl acetate or phosphotungstic acid) that provides high contrast, so vesicles can be viewed against a dark-stained background (Ruozi et al., 2011). The negative-staining technique is relatively fast and simple; however, it has been shown to cause changes to the original liposome structure and can lead to the creation of light and dark fringes that may be mistaken for lamellar structures (Bibi et al., 2011; Ruozi et al., 2011). In addition, placing the sample under vacuum can cause further dehydration of the sample, which can again cause changes in the structure of the vesicles (Bibi et al., 2011). Whilst the negative-stain TEM technique can provide much higher magnification for imaging nanoparticles, the damage to the liposome structure makes it difficult to accurately evaluate the morphological characteristics of the sample. Therefore, other imaging techniques are generally required to confirm results.

#### Freeze–Fracture Technique

The freeze–fracture technique does not require any drying process and can provide additional information about the internal structure of nanoparticles (Kuntsche et al., 2011). This method involves placing a sample on a TEM grid that is sandwiched between two copper or gold holders (Severs, 2007). The sample is vitrified via rapid freezing, typically with liquid propane or liquid nitrogen, before being fractured along areas of the sample with weak molecular interactions. This fracture surface can be further etched and shadowed with a thin platinum or carbon layer to provide a “negative” replica of the fracture sample plane (Severs, 2007; Kuntsche et al., 2011). The replica is then cleaned with an organic solvent to remove all residues prior to visualization under a TEM microscope. As these replicas are so stable, they can be stored and viewed later (Kuntsche et al., 2011). The major advantage of this technique is that the replicas closely reflect the native state of the sample, and can provide detailed information on the 3D structure of the vesicles and bilayer organization (Bibi et al., 2011; Kuntsche et al., 2011). This information includes the multilamellar construction and bilayer packing of multilamellar vesicles. This technique can also assess aggregate size and may be particularly useful for examining the interaction of cationic liposomes with DNA (Bibi et al., 2011). However, artifacts may still occur in the sample during preparation due to insufficient freezing rate, re-deposition of solvent molecules and/or mechanical stress (Severs, 2007; Kuntsche et al., 2011). For example, a “rippling effect” can occur on the SUVs in the sample (Bibi et al., 2011). This is a common bilayer deformation that is due to a disorder in the transitions of the acyl chains prior to freezing. Incubating vesicles between the pre-transition and actual transition temperature can also cause ripples (Bibi et al., 2011). Interestingly, this can be used to provide information into the lipid phase transitions that occur with the varying nature of lipids used for liposomal preparations (Bibi et al., 2011).

#### Cryogenic TEM

Cryogenic TEM (cryo-TEM) is a variation of TEM where thin aqueous hydrated films which are vitrified in liquid ethane are used prior to imaging. This technique allows for the analysis of liposomes in their most native state, and is a valuable tool to determine the size, shape, internal structure, and lamellarity of liposomes (Weisman et al., 2004; Bibi et al., 2011; Kuntsche et al., 2011). The major advantage of rapidly freezing liposome samples is minimizing the formation of ice crystals and preserving proteins or other materials (Bibi et al., 2011; Kuntsche et al., 2011). This is beneficial where proteins or DNA have been encapsulated within the nanoparticles (Weisman et al., 2004; Kuntsche et al., 2011). Cryo-TEM is the most useful form of microscopy currently available to study liposomes, as it avoids issues with chemical fixation, dehydration, cutting and staining – all of which can affect the morphology of vesicles (Bibi et al., 2011). The resolution range is ∼5 to 500 nm, as defined by the thickness of the film (Almgren et al., 2000). However, several limitations should be noted with this technique, including the fact that only a 2D image is obtained from 3D objects (which generally also applies to most of the other microscopy techniques). To overcome this limitation, reconstruction of 3D shapes from a sufficiently large number of 2D images of randomly oriented non-spherical particles is possible (Orlova et al., 1999), as well as viewing the sample at different tilt angles to attain information about 3D shape (Van Antwerpen and Gilkey, 1994). Cryogenic electron tomography (cryo-ET) can also be used instead to attain 3D images (Le Bihan et al., 2009). Cryo-TEM also utilizes lower doses of electrons, which means that it often has a lower resolution compared to other methods (Bibi et al., 2011; Kuntsche et al., 2011). Artifacts are still possible due to the formation of a thin film of amorphous ice and the use of blotting on the sample applying shear forces during the film formation (Almgren et al., 2000; Bibi et al., 2011).

### Environmental Scanning Electron Microscopy (ESEM)

Environmental scanning electron microscopy (ESEM) is an imaging system that does not require the use of fixing, staining or freezing of vesicles, and can allow imaging of dynamic changes of wet systems without previous sample preparation (Muscariello et al., 2005; Ruozi et al., 2011). The main feature of ESEM is the presence of water vapor in the microscope chamber. The ability to maintain a water-containing atmosphere around the sample that may be partially or even fully hydrated is made possible by the use of a multiple-aperture, graduated vacuum system that allows the imaging chamber to be sustained at pressures up to 55 hPa (Bibi et al., 2011; Ruozi et al., 2011). The primary electron beam can generate secondary electrons that then encounter vapor molecules, leading to a cascade amplification of the signal before reaching the detector. Because of this, ESEM does not require sample preparation (Muscariello et al., 2005). This technique allows for variation in the sample environment through a series of pressure, temperature and gas compositions (Mohammed et al., 2004), which is useful when determining how environmental changes affect the vesicles. This is applicable to nanopharmaceutical formulation and stability studies (Bibi et al., 2011). ESEM has also been used to analyze drug loading into the bilayer of liposomes (Mohammed et al., 2004), as well as determining the size and shape of vesicles. A limitation of ESEM is that it cannot provide detailed information regarding the lamellarity and internal architecture of the nanoscale structures (Ruozi et al., 2011).

## Atomic Force Microscopy (AFM)

Atomic force microscopy (AFM), also known as scanning-force microscopy (SFM), is a type of scanning probe microscope technique. It works by running a sharp tip attached to a cantilever and sensor over the surface of a sample and measuring the surface forces between the probe and the sample (Sitterberg et al., 2010). As the cantilever runs along the sample surface, it moves up and down due to the surface features and the cantilever deflects accordingly. This deflection is usually quantified using an optical sensor, with the laser beam being reflected on the back of the cantilever onto the light detector (Sitterberg et al., 2010). AFM does not need to operate in a vacuum and can operate in ambient air or under liquid; hence it is increasingly being used to image biological samples as well as nanoparticles (Liang et al., 2004a,b; Ruozi et al., 2005, 2009). AFM has outstanding resolution in the order of fractions of a nanometer and can provide a 3D image of liposomes along with details on morphology, size distribution, homogeneity, and stability (Liang et al., 2004a,b; Ruozi et al., 2005, 2007, 2009). Importantly, AFM can be used to characterize the surface modifications of liposomes and detect ligands (e.g., antibodies and polymers) conjugated at the liposomal membrane surface (Bendas et al., 1999; Moutardier et al., 2003; Anabousi et al., 2005; Liang et al., 2005). For example, Bendas et al. used AFM to magnify the liposomal membrane border and were able to image trimeric structures, approximately 8–10 nm in diameter, which represented the coupling of IgG antibodies to the liposome surface (Bendas et al., 1999). The findings showed that the effectiveness of the technique was highly dependent on the conjugation method used, with antibodies conjugated directly to the liposomal surface being visible with AFM (restricted protein mobility), in comparison to antibodies attached to PEG chains. It was suggestive that the PEG chains caused high protein mobility and, therefore, were unable to be scanned (Bendas et al., 1999). Conversely, Anabousi et al showed that incorporation of PEGylated lipids into the liposomes induced a steric stabilization with liposomes maintaining a spherical shape (Anabousi et al., 2005). AFM images of the surface of unconjugated liposomes were smooth and no structures could be observed, whereas conjugation of transferrin to the surface of PEGylated liposomes were visualized as small globular structures (Anabousi et al., 2005). Similarly, Moutardier et al manufactured liposomes with polymeric cores (LSP) that consisted of drugs loaded into polymeric particles that formed the core of lipid vesicles (Moutardier et al., 2003). Images taken using AFM showed the presence of a polymer network on the exterior surface, which suggested that the collagen polymeric core radiated out and formed a surface layer on the LSP (Moutardier et al., 2003). In addition to surface structural details, AFM can provide information about the mechanical and chemical properties of a sample surface through force measurements (Ruozi et al., 2007). For example, Mao et al. used this technique to assess the elasticity and adhesive properties of liposomes (Mao et al., 2004). One limitation of AFM is the need for nanoparticles to be adsorbed onto support surfaces, such as mica or silicon wafers. The adsorption of liposomes onto a solid substrate has the potential to modify the size and shape of the vesicles, and cause their flattening. (Ruozi et al., 2007). In addition, the periodic contact of the probing tip can drag the liposomes as it moves across the vesicles in a sample (Jass et al., 2000). Despite this, AFM is still a useful tool in the evaluation of liposomes.

## Conclusion

There is a wide range of imaging techniques available for evaluating the morphology of liposomes, with each having its own advantages and disadvantages. Light microscopy can provide general details regarding the size and shape of larger vesicles and the homogeneity of a sample in a relatively fast manner. Conversely, TEM is the most commonly used technique to examine the morphology of liposomes in much more detail; however, the potential for structural changes with each TEM sub-type due to staining and/or exposure to vacuum conditions need to be considered when interpreting the results. ESEM is most useful when determining liposomal changes in response to the environment, whereas AFM is emerging as a useful method in the morphological analysis of nanoparticles and provides maximum resolution of the liposomal surface. Both AFM and TEM are capable of imaging ligands conjugated to the surface of liposomes and provide complementary information on surface modifications. Overall, the choice of technique is dependent on what morphological characteristics and degree of detail are required. In addition, understanding the potential effects of the sample preparation method of each imaging technique is important in the selection process.

## Author Contributions

Drafting of manuscript: A-LR and SH. Revising the article critically for important intellectual content: SH, PD, JF, WP, AM, DS and AW.

## Conflict of Interest Statement

The authors declare that the research was conducted in the absence of any commercial or financial relationships that could be construed as a potential conflict of interest.
